# Product Aspect Clustering by Incorporating Background Knowledge for Opinion Mining

**DOI:** 10.1371/journal.pone.0159901

**Published:** 2016-08-25

**Authors:** Yiheng Chen, Yanyan Zhao, Bing Qin, Ting Liu

**Affiliations:** 1 Department of Computer Science and Technology, Harbin Institute of Technology, Harbin, China; 2 Department of Media Technology and Art, Harbin Institute of Technology, Harbin, China; Jiangnan University, CHINA

## Abstract

Product aspect recognition is a key task in fine-grained opinion mining. Current methods primarily focus on the extraction of aspects from the product reviews. However, it is also important to cluster synonymous extracted aspects into the same category. In this paper, we focus on the problem of product aspect clustering. The primary challenge is to properly cluster and generalize aspects that have similar meanings but different representations. To address this problem, we learn two types of background knowledge for each extracted aspect based on two types of effective aspect relations: relevant aspect relations and irrelevant aspect relations, which describe two different types of relationships between two aspects. Based on these two types of relationships, we can assign many relevant and irrelevant aspects into two different sets as the background knowledge to describe each product aspect. To obtain abundant background knowledge for each product aspect, we can enrich the available information with background knowledge from the Web. Then, we design a hierarchical clustering algorithm to cluster these aspects into different groups, in which aspect similarity is computed using the relevant and irrelevant aspect sets for each product aspect. Experimental results obtained in both camera and mobile phone domains demonstrate that the proposed product aspect clustering method based on two types of background knowledge performs better than the baseline approach without the use of background knowledge. Moreover, the experimental results also indicate that expanding the available background knowledge using the Web is feasible.

## Introduction

Social media holds a considerable amount of user-generated content describing the opinions of customers on products and services in the forms of reviews, blog posts, tweets, etc. These reviews are valuable for customers to make purchasing decisions and for companies to guide the business activities. Consequently, the advent of social media has stirred considerable excitement and provided abundant opportunities for opinion mining and sentiment analysis [1–3].

Opinion mining and sentiment analysis entail a number of interesting and challenging tasks, such as sentiment classification [2, 4, 5], sentiment extraction [6–10] and sentiment summarization [11–13]. One fundamental task that is necessary for fine-grained opinion mining is aspect recognition [7, 8, 14–16] with the purpose of identifying the main topic addressed in a review. In many practical opinion mining applications, such as opinion summarization [13, 17] and recommender systems [18, 19], product aspect recognition is always treated as the first step.

Aspect recognition consists of two sub-tasks. One is aspect extraction, and the other is aspect clustering. The purpose of aspect extraction is to extract the entities on which the users are commenting. For example, this task may involve the extraction of “图像” (“picture” in English) as the product aspect from the review sentence “图像很漂亮” (“the picture is great”). Meanwhile, the purpose of aspect clustering is to cluster aspects that have similar meanings into the same groups. For example, the words “图像” (“picture” in English) and “照片” (“photo” in English) express the same meaning and therefore, we need to group them.

Aspect extraction and aspect clustering are both critical for many opinion mining applications. To date, most of the relevant research work on aspect recognition has concentrated on the first sub-task. Many types of methods have been proposed, including rule-based [14, 20–22], supervised [23–25], and topic-model-based [8, 26, 27] methods, for the extraction of aspects. However, only a few studies have been performed on aspect clustering.

It can be observed that people commonly use different words or phrases to express the same aspect of a product. Obviously, for a single product, it is very important to cluster these words or phrases into the same group to produce a more accurate summary. Moreover, product aspect clustering is also a necessary step of domain ontology construction. Because of the importance of the aspect clustering task, it deserves greater attention. This paper is primarily focusing on this task. In previous work, several researchers have used topic-model-based methods [8, 11, 28] to cluster domain-specific aspects. However, topic models always jointly model topics and sentiment words. Certain researchers treat this task as a traditional clustering task, the key element of which is the similarity computation. For example, Zhai et al. [29] modeled this task as a semi-supervised learning problem using lexical similarity. However, their method requires several manually selected seeds as input. Because the seeds are random, it is correspondingly difficult to implement or experimentally reproduce this method.

In this paper, we also treat this task as a typical clustering task that can be solved using many existing clustering methods. Therefore, the computation of the similarity between two aspects is regarded as the key technique. The primary obstacle is to determine how to approximately cluster and generalize aspects that have similar meanings but different representations. Obviously, the commonly used similarity measures that are based on the literal meanings of two aspects are far from sufficient. To address this problem, in this paper, we propose a simple and effective unsupervised method that can incorporate a large amount of cross-document background knowledge on each extracted aspect. Thus, the rich background knowledge that is available for each aspect allows them to be better grouped.

Specifically, this background knowledge is captured primarily on the basis of two types of effective aspect relations. One type is a **relevant aspect relation**, which indicates that the two aspects are relevant to each other and must be grouped. For example, the aspects “人像镜头” (“portrait lens” in English) and “镜头” (“lens” in English) are relevant to each other, exhibiting a relevant aspect relation. The other type of relation that is used to enrich the background knowledge available for each aspect is an **irrelevant aspect relation**. This type of relation indicates that two aspects are irrelevant to each other and must not be grouped. For example, the aspects “照片” and aspect “分辨率” are distinct; they are irrelevant to each other, so they must not be grouped.

Based on these two types of aspect relations, we can construct both a relevant aspect set and an irrelevant aspect set from a large review corpus to serve as the cross-document background knowledge for each product aspect. In other words, we can obtain two types of background knowledge for each aspect. On the one hand, the relevant aspect set supports the attempt to more accurately obtain the domain synonyms for a given aspect. On the other hand, the irrelevant aspect set helps to separate this given aspect from other irrelevant aspects with which it should not be grouped. Thus, the main emphasis of our research has shifted to the exploitation of these two sets of aspects for each extracted aspect. To obtain more background knowledge to enrich each aspect set, in this paper, we attempt to exploit such knowledge both from a large review corpus and from the Web.

Because aspect clustering is a typical clustering problem, a hierarchical clustering method is applied in this paper to classify aspects into different groups based on their associated cross-document background knowledge, namely, the relevant and irrelevant aspect sets. Several similarity computation methods, including literal similarity, relevant set similarity, and irrelevant set similarity, are designed for the computation of the similarity between two aspects.

In summary, this paper makes the following contributions:

1. It proposes the exploitation of a relevant aspect set and an irrelevant aspect set to supplement the background knowledge available for each extracted aspect. This process is fully automatic.
2. It proposes several effective similarity computation methods based on the above two types of cross-document background knowledge. A hierarchical clustering method is then applied using these methods.
3. It proposes an effective method of learning these two types of background knowledge from a large dataset. To obtain more knowledge, we expand them from the Web.
4. We evaluate our method on the corpora of camera and mobile phone domains as a case study. The experimental results show that using both types of background knowledge yields significant performances that gain over the baseline clustering method without using the knowledge. Furthermore, the two types of knowledge are complementary to each other.

The remainder of this paper is organized as follows. Section 2 introduces the two types of background knowledge based on the two aspect relations. Section 3 presents the hierarchical clustering algorithm based on the two types of background knowledge. Section 4 presents the experiments and results. Section 5 summarizes the related work. Finally, we conclude this paper in Section 6.

This paper is a substantial extension of our earlier work in [30]. In this paper, we improve the method in [30] by adding a background knowledge expansion step. We treat the Web as a large corpus and expand much more relevant and irrelevant aspect sets from the Web to expand the background knowledge; furthermore, we annotate additional data of mobile phone domain to better evaluate the framework of incorporating background knowledge for aspect clustering task, and we add several new experimental results, especially on using the method of expanding rich background knowledge from Web and using the corpora from two product domains to demonstrate the effectiveness of our framework. Moreover, a comprehensive description of our method, and an in-depth analysis of the results are included in this paper.

## Learning Background Knowledge

We consider two types of background knowledge for each aspect. One is the set of relevant aspects, which is constructed based on relevant aspect relations. The other is the set of irrelevant aspects, which is constructed based on irrelevant aspect relations. In this section, we first present the definitions of relevant aspect relations and irrelevant aspect relations. Then, we discuss how to extract the relevant and irrelevant aspect sets for each aspect. Finally, we discuss how to learn more background knowledge.

### Definition

**Relevant aspect relation** refers to the relation between two relevant aspects. Two aspects that satisfy this type of relation can be grouped.

We can observe a ***relevant aspect phenomenon*** as follows. *In a given sentence, if one aspect contains another, then the two aspects are relevant and can be grouped into the same cluster*. For example, in the sentence “我今天买了个[人像镜头], 这个[镜头]的分辨率非常不错啊” (“I bought a [portrait lens], and the resolution of this [lens] is perfect”), the aspects “人像镜头” (“portrait lens” in English) and “镜头” (“lens” in English) exhibit relevant aspect phenomenon, indicating a relevant aspect relation. Thus, the two aspects can be classified into the same group.

**Irrelevant aspect relation** refers to the relation between two irrelevant aspects. Two aspects that satisfy this type of relation must not be grouped.

We can observe an ***irrelevant aspect phenomenon*** as follows. *In a given sentence, the product aspect of interest is always used in one particular form instead of different forms, even though this aspect can be expressed in other forms*. Based on this phenomenon, aspects that appear in the same sentence can be regarded as different aspects if they do not contain each other.

Consider the following two sentences as an example:

- Sentence 1: 我在电脑上浏览佳能600D的[照片]时, 感觉[照片]挺不错的, <分辨率>挺高∘
  (I browsed the [*pictures*] on the computer, and I found that the [*pictures*] were perfect and the <*resolution*> was high.)
- Sentence 2: 我在电脑上浏览佳能600D的[照片]时, 感觉[图像]挺不错的, <分辨率>挺高∘
  (I browsed the [*pictures*] on the computer, and I found that the [*photos*] were perfect and the <*resolution*> was high.)

In most cases, if a word is to be mentioned multiple times in the same sentence, people will tend to always use the same word. For instance, in Sentence 1, the word “照片” (“picture” in English) appears twice and is not expressed in any other form. By contrast, we seldom use different word representations to express the same meaning in the same sentence, such as the words “照片” (“picture” in English) and “图像” (“photo” in English) used in Sentence 2. Based on these considerations, in Sentence 1, because the aspect “照片” and the aspect “分辨率” do not contain each other, they can be regarded as different aspects; in other words, they belong to different groups. Thus, the relationship between them is recorded as an irrelevant aspect relation.

### How to Learn

According to the definitions of *relevant* and *irrelevant aspect relations*, two types of aspect sets, namely, a relevant aspect set and an irrelevant aspect set can be constructed for each aspect. An example of a Chinese review that is tagged with all product aspects that appear in it, is shown as follows.

Example:

镜头采用了专业的施奈德3倍**光变镜头**, 光圈为F2.8–F4.8, 虽然指标并不出众, 但是专业的镜头相对来说会给成像效果带来相当大的助益;

Translation as:

The lens is the professional Schneider 3x **optical zoom lens**, with an aperture between F2.8 and F4.8; although these performance indicators are not outstanding, this professional lens is reasonably beneficial to the image quality.

In the example, four product aspects can be extracted. Consider the aspect “镜头” (“lens” in English) as an example; because “镜头” is the suffix of “光变镜头” (“optical zoom lens”), “光变镜头” is an aspect that is relevant to the considerable aspect “镜头” and can be added to the relevant set. By contrast, “光圈” and “成像效果” are completely different from “镜头” in literal meaning; thus, they are added to the irrelevant set.

Formally, we describe each aspect *a* using a tuple <*set_R*, *set_IR*>, where *set_R* is the set that stores items relevant to *a* and *set_IR* is the set that stores items irrelevant to *a*. Thus, the background knowledge for aspect *a* can be represented as follows:

$$\begin{array}{ccc} & & a:set\_R[r_{1},r_{2},...,r_{i},...,r_{n}]\\ & & \;set\_IR[ir_{1},ir_{2},...,ir_{j},...,ir_{m}] \end{array}$$

Here, *r*_*i*_ is an aspect that is relevant to the aspect *a* and *ir*_*j*_ is an aspect that is irrelevant to the aspect *a*; *n* represents the number of relevant aspects, and *m* represents the number of irrelevant aspects. Based on observations of the two phenomena described above in Section 2.1, we can follow the steps below to obtain the relevant and irrelevant aspects from a sentence in which the aspect *a* appears.

- **relevant aspect *r*_*i*_**: the aspects *a* and *r*_*i*_ are relevant to each other, if *a* and *r*_*i*_ appear in the same sentence and exhibit an inclusion relation, e.g., *a* is the suffix of *r*_*i*_ or vice versa.
- **irrelevant aspect *ir*_*j*_**: the aspects *a* and *ir*_*j*_ are irrelevant to each other, if *a* and *ir*_*j*_ appear in the same sentence and do not exhibit an inclusion relation with each other.

As a result, if we use only the background knowledge obtained from this sentence in which the aspect appears, the background knowledge for “镜头” in the above example can be expressed as follows:

$$\begin{array}{ccc} & & \;镜头:set\_R\;[光变镜头]\\ & & \;\;set\_IR[光圈,成像效果]\\ & & \left(\text{lens}:set\_R\;[\text{optical}\;\text{zoom}\;\text{lens}\right]\\ & & \;set\_IR[\text{aperture,}\;\text{image}\;\text{quality}]\\ & & \;\;\text{in}\;\text{English}). \end{array}$$

Here, “光变镜头” is a relevant aspect, and “光圈” and “成像效果” are irrelevant aspects. However, it is evident that the background knowledge captured from only one sentence is limited. We require more knowledge. Because an aspect such as “镜头” may appear in many review sentences, we can accordingly acquire large numbers of relevant and irrelevant aspects from a domain-specific corpus. Then, a final *set_R* and *set_IR* with more aspect elements can be constructed. We summarize the detailed algorithm for this set acquisition procedure in Algorithm 1.

For example, for the aspect “镜头”, 71 relevant aspects and 149 irrelevant aspects can be captured from a camera domain corpus containing 138 reviews. Based on the background knowledge gathered in this way, we can design new hierarchical clustering algorithms for clustering product aspects into differentferent groups.

**Algorithm 1**: Relevant and Irrelevant Aspect Set Acquisition Algorithm

 **input**: A product aspect *a* and a corpus *C*

 **output**: The relevant aspect set *R* and the irrelevant aspect set *IR*

 **for** *each sentence s in C* **do**

  **if** *s contains a* **then**

   **for** *each aspect* *a*_*i*_ *in s* **do**

    **if** *there is an inclusion relation between a and* *a*_*i*_ **then**

     *R* = *R*⋃{*a*_*i*_}

    **else**

     *IR* = *IR*⋃{*a*_*i*_}

To estimate the effectiveness of this method of extracting the relevant/irrelevant sets for each aspect, we labeled all aspects appearing in 40 reviews in the camera domain. We were thus able to build 1,053 tuples representing aspects, such as <*a*, *r*_*i*_> or <*a*, *ir*_*j*_>, based on the two types of aspect relations discussed above. We manually evaluated each tuple and observed that the accuracy was 99.34%, representing significant performance. In other words, the algorithm presented in Algorithm 1 is a valid means of obtaining the relevant and irrelevant sets for an aspect.

As a result, for a product aspect *a*, the relevant and irrelevant aspect sets obtained based on the two corresponding types of relations between aspects can be regarded as standard background knowledge with which to enrich the semantic meaning of the aspect itself. Furthermore, they can be effectively used in subsequent aspect clustering.

### How to Learn More

In theory, for a given aspect, the availability of more background knowledge makes it easier to distinguish that aspect from other aspects in the same domain. However, because a review corpus is necessarily limited, the background knowledge for each aspect that can be learned from such a corpus is also limited. To address this problem, we can treat the Web as a large corpus and expand the relevant and irrelevant aspect sets based on information obtained from the Web to expand our captured knowledge.

Our algorithm begins by searching for the aspect of interest as the query using Baidu. It can return lots of snippets containing the given aspect. In other words, it can increase the number of available sentences containing the queried aspect compared with the number of relevant sentences that can be obtained from a corpus. As a result, the algorithm summarized in Algorithm 1 can capture more relevant and irrelevant aspects when applied to the sentences retrieved from the Web.

## Hierarchical Clustering by Incorporating the Background Knowledge

Although how to learn the two types of background knowledge and how to learn more knowledge are the key parts of our method, how to incorporate the two kinds of background knowledge is another challenge that needs to be solved. It is obvious that the aspect clustering is a typical clustering task, and we can use a clustering algorithm to incorporate the two types of background knowledge. It is known that clustering research and application has a long history. Over the years, a vast collection of clustering algorithms has been designed [31–33]. In this paper, we choose the hierarchical clustering algorithm, because it is simple and effective, and meanwhile has been widely used in previous studies. More importantly, the hierarchical clustering algorithm can better incorporate the two types of background knowledge. We can certainly try other available clustering methods, such as the k-means clustering algorithm in the future.

Similarity computation between two aspects is the key task in the hierarchical clustering algorithm. Traditional similarity computation measures typically use thesauri or simply compute the literal similarity between two aspects. However, these methods are far from sufficient for several reasons.

First, many product aspects are domain-related words or phrases, which are not included in traditional thesauri. For example, the aspect “光变镜头” (“optical zoom lens” in English) does not appear in any dictionary. Second, many aspects are not dictionary synonyms, but indicate the same aspect in a particular context or domain, such as the aspects “照片” (“photo” in English) and “片子” (“photo” in English) in the camera domain.

To address these problems, Section 2 introduces the acquisition of cross-document background knowledge for each aspect, including relevant and irrelevant aspect sets. With this approach, we can use additional knowledge, beyond the limited literal evidence (knowledge), to compute the similarity between two aspects by incorporating these two sets.

Accordingly, in this paper we design two types of similarity computation methods. One involves computation based on the relevant sets. The other involves computation based on the irrelevant sets. In combination with the previously established literal computation method, the computation of the similarity between two aspects *a*_*i*_ and *a*_*j*_ comprises three components:

- Literal Similarity (LS): refers to the similarity between *a*_*i*_ and *a*_*j*_ in the literal sense, which is denoted by *s*_1_(*a*_*i*_, *a*_*j*_). In this component, two factors are considered. One is an exploration of whether these two aspects are synonyms according to their dictionary definitions. The other is the literal computation of the similarity between *a*_*i*_ and *a*_*j*_. In other words, we treat each character as an element; then, each aspect can be regarded as a vector of characters. Many similarity methods can be used. In this paper, we simply consider the Cosine similarity measure as a representative example. Based on these two factors, we express this type of similarity as follows:
  $$\begin{array}{c} s_{1}\left(a_{i},a_{j}\right)=\{\begin{array}{cc} 1 & \text{if}\;a_{i}\;\text{and}\;a_{j}\;\text{are}\;\\ & \;\;\;\;\;\;\;\text{synonyms,}\\ \cos(a_{i},a_{j}) & \text{if}\;a_{i}\;\text{and}\;a_{j}\;\text{are}\\ & \;\text{not}\;\text{synonyms.} \end{array} \end{array}$$ (1)
- Relevant Set Similarity (RSS): refers to the similarity between the relevant aspect sets of *a*_*i*_ and *a*_*j*_, which is denoted by *s*_2_(*a*_*i*_, *a*_*j*_). This concept is based on the hypothesis that the relevant aspect sets of two similar aspects should also be similar. In other words, when two relevant aspect sets are similar, it indicates that their corresponding aspects should tend to be grouped. Because the relevant background knowledge for each aspect can be expressed as a vector, the computation of the relevant set similarity can be converted into a computation of the similarity between the two vectors thus constructed. The computation procedure is shown as follows.
  $$\begin{array}{ccc} s_{2}\left(a_{i},a_{j}\right) & = & sim(rel\_vector_{i},rel\_vector_{j})\\ & = & \frac{rel\_vector_{i}\cdot rel\_vector_{j}}{∥rel\_vector_{i}∥∥rel\_vector_{j}∥} \end{array}$$ (2)
- IRrelevant Set Similarity (IRSS): refers to the similarity between the irrelevant aspect sets of *a*_*i*_ and *a*_*j*_, which is denoted by *s*_3_(*a*_*i*_, *a*_*j*_). This similarity is computed based on the hypothesis that if *a*_*i*_ is similar to *a*_*j*_, then it cannot appear in the irrelevant aspect set of *a*_*j*_. In other words, if *a*_*i*_ appears in the irrelevant aspect set of *a*_*j*_, this indicates that *a*_*i*_ and *a*_*j*_ should not be grouped. We describe the similarity between *a*_*i*_ and *a*_*j*_ as follows:
  $$\begin{array}{c} s_{3}\left(a_{i},a_{j}\right)=\{\begin{array}{cc} 1 & \text{if}\;a_{i}\;\text{appears}\;\text{in}\;\text{the}\;\text{irrelevant}\\ & \text{aspect}\;\text{set}\;\text{of}\;a_{j}\text{,}\\ 1 & \text{if}\;a_{j}\;\text{appears}\;\text{in}\;\text{the}\;\text{irrelevant}\\ & \text{aspect}\;\text{set}\;\text{of}\;a_{i}\text{,}\\ 0 & \text{else.} \end{array} \end{array}$$ (3)

The above three methods of similarity computation can reflect three different perspectives on similarity. We can combine them to produce a more effective method of similarity computation. In order to better describe the relations among the three kinds of similarities, we adopt a linear combination method. The linear combination can be considered as linear interpolation, which is a standard method to fuse multiple features (perspectives). This kind of method is flexible and simple to adjust combination coefficients to match an object. More formally, the final similarity between aspects *a*_*i*_ and *a*_*j*_ can be expressed as follows:

$$\begin{array}{ccc} S_{a}\left(a_{i},a_{j}\right) & = & (\alpha *s_{1}\left(a_{i},a_{j}\right)+\beta *s_{2}\left(a_{i},a_{j}\right)\\ & & -\gamma *s_{3}\left(a_{i},a_{j}\right)) \end{array}$$ (4)

where the similarity *s*_1_ reflects the literal computation, *s*_2_ reflects the relevant aspect phenomenon and *s*_3_ reflects the irrelevant aspect phenomenon. A hierarchical clustering algorithm based on this similarity measure is described in detail in Algorithm 2.

**Algorithm 2**: Hierarchical Clustering Algorithm based on the New Similarity Measure

 **input**: Set of aspects *A*, $A=\{a_{1},a_{2},\ldots ,a_{n}\}$; each aspect is described by *R* and *IR*

 **output**: Aspect clusters *AC*

 1. Define each aspect as a cluster, denoted by $c_{1},\ldots ,c_{i},\ldots ,c_{n}$;

 2. Compute the similarity between each pair of clusters;

 **if** *the similarity between* *c*_*i*_ *and* *c*_*j*_ *is maximum and greater than* *θ* **then**

  Merge *c*_*i*_ and *c*_*j*_ into a new cluster

 3. Repeat 2 until the number of clusters does not change;

 4. The final clusters are *AC*;

Here, in Step 2, the similarity between two clusters $c_{i}=\left\{a_{1}^{i},...,a_{p}^{i},...,a_{n}^{i}\right\}$ and $c_{j}=\left\{a_{1}^{j},...,a_{q}^{j},...,a_{m}^{j}\right\}$ is computed as follows.

$$\begin{array}{c} S_{c}\left(c_{i},c_{j}\right)=\frac{\sum_{p=1}^{n}\sum_{q=1}^{m}S_{a}\left(a_{p}^{i},a_{q}^{j}\right)}{n\times m} \end{array}$$ (5)

## Experiments

### Experimental Setup

#### Corpus

We conducted experiments on the Chinese corpora in the digital camera and mobile phone domains that were dawn from the corpora of the Chinese Opinion Analysis Evaluation 2008 (COAE 2008). Table 1 provides the detailed statistics of the corpora.

**Table 1:** Corpora statistics from the digital camera and mobile phone domains.

Statistics	Camera Domain	Mobile Phone Domain
# reviews	138	123
# aspects (before reduplication removing)	4,039	1,490
# aspects (after reduplication removing)	1,189	757
# single aspects	867	574
# multiple aspects	322	183
average # per aspect	3.4	2.0

The corpus of the camera domain contains 138 reviews, in which 4,039 aspects were manually identified and annotated before the removal of duplications and 1,189 aspects remained after duplication removal. The corpus of the mobile phone domain contains 123 reviews, in which 1,490 aspects were manually identified and annotated before the removal of duplications and 757 aspects remained after duplication removal. This table indicates that each aspect appeared approximately 3.4 times on average for camera domain and 2.0 times for mobile phone domain. Therefore, for each aspect, we were able to collect relevant and irrelevant aspect sets from many review sentences.

#### Evaluation

We used the classic clustering evaluation metrics, *Entropy* and *Purity* [29, 34, 35] to evaluate the results of the aspect clustering task in this study. Given a data set *DS*, its gold partition is *G* = *g*_1_, …, *g*_*j*_, …, *g*_*k*_, where *k* is the given number of clusters. Suppose that our background knowledge based method can group *DS* into *k* disjoint subsets, that is, *DS* = *DS*_1_, …, *DS*_*i*_, …, *DS*_*k*_; then, *Entropy* and *Purity* can be defined as follows.

**Entropy**: For each resulting cluster *DS*_*i*_, we can quantify its entropy using Eq (6), where *P*_*i*_(*g*_*j*_) is the proportion of *g*_*j*_ data points in *DS*_*i*_. The total entropy of the overall clustering result (considering all clusters) is calculated using Eq (7).

$$\begin{array}{c} entropy\left(DS_{i}\right)=-\sum_{j=1}^{k}P_{i}\left(g_{j}\right)\log_{2}P_{i}\left(g_{j}\right) \end{array}$$ (6)

$$\begin{array}{c} entropy_{total}=\sum_{i=1}^{k}\frac{|DS_{i}|}{\left|DS\right|}entropy\left(DS_{i}\right) \end{array}$$ (7)

**Purity**: Purity measures the extent to which that a cluster contains only data from one gold-partition. The cluster purity is computed using Eq (8). The total purity of the overall clustering result (all clusters) is computed using Eq (9).

$$\begin{array}{c} purity\left(DS_{i}\right)=\max_{j}P_{i}\left(g_{j}\right) \end{array}$$ (8)

$$\begin{array}{c} purity_{total}=\sum_{i=1}^{k}\frac{|DS_{i}|}{\left|DS\right|}purity\left(DS_{i}\right) \end{array}$$ (9)

According to the definition of purity, high purity is easy to achieve when the number of clusters is large—in particular, purity is 1 if each aspect gets its own cluster. Thus, we need to use the measure “entropy” to trade off the quality of the clustering against the number of clusters. Entropy that is from information theory, refers to the expected value (average) of the information contained in each message.

Based on theses, we need to simultaneously use the evaluation metrics “Entropy” and “Purity” to evaluate the final clustering results. The system with lower entropy value and higher purity value performs better.

#### Comparative systems

As mentioned above, the computation of the similarity between two aspects is the primary challenge in the aspect clustering procedure. Based on the background knowledge learned as described in Section 2 and the three measures of the similarity between two aspects summarized in Section 3, we designed four systems for comparison to demonstrate the performance of each similarity measure for aspect clustering.

- Literal Similarity (LS): We consider only the literal meaning of each aspect. We compute the similarity between two aspects *a*_*i*_ and *a*_*j*_ literally.
- Relevant Set Similarity (RSS) + LS: We consider the relevance relations between aspects. We compute the similarity between two aspects *a*_*i*_ and *a*_*j*_ using their relevant aspect sets, on the basis of the literal similarity.
- IRrelevant Set Similarity (IRSS) + LS: We consider the irrelevance relations between aspects. We compute the similarity between two aspects *a*_*i*_ and *a*_*j*_ using their irrelevant aspect sets, on the basis of the literal similarity.
- RSS + IRSS + LS: We combine the three types of similarities between the two aspects *a*_*i*_ and *a*_*j*_ to obtain a final similarity. This approach also represents the method proposed in this paper.

### Results

We conducted two experiments. The purpose of the first was to confirm the effectiveness of using our proposed two types of background knowledge in the aspect clustering task. In detail, we also apply this kind of framework into two product domains, i.e., digital camera and mobile phone domains, to demonstrate this framework can be portable to different product domains. The purpose of the other was to prove whether the attempt to enrich the available background knowledge using the Web is effective.

In our method, we need to tune three parameters, *α*, *β* and *γ* in Eq (4). Generally speaking, parameter optimization is the problem of choosing a set of parameters for a learning algorithm, usually with the goal of optimizing a measure of the algorithm’s performance on an independent data set. The traditional way of performing parameter optimization has been grid search, or a parameter sweep, which is simply an exhaustive searching through a manually specified subset of the parameter space of a learning algorithm. A grid search algorithm must be guided by some performance metric, typically measured by cross-validation on the training set or evaluation on a held-out validation set.

Because we just have three parameters to be optimized in this paper, we choose the grid search method to optimize the parameters *α*, *β* and *γ* in Eq (4). In detail, we annotate additional sets of reviews for the camera and mobile phone domains respectively as the training data. Then, we apply an exhaustive searching through a specified subset of the parameter space [0.1, 0.2, 0.3, 0.4, 0.5, 0.6, 0.7, 0.8, 0.9, 1.0]. That is to say, the three parameters *α*, *β*, *γ* can be assigned the values from this space. Finally, measured by the Entropy and Purity methods, we can obtain the optimal parameters, that is, *α* = 0.5, *β* = 0.5, and *γ* = 1.0 for both domains.

#### Effectiveness of the Use of Background Knowledge in Aspect Clustering

Table 2 shows the experimental results of the four systems in the product aspect clustering task. The four systems differ because of the differing background knowledge they use in the aspect similarity computation.

**Table 2:** Comparisons of the results obtained using different similarity computation measures in the product aspect clustering task for the camera and mobile phone domains.

Method	Camera Domain		Mobile Phone Domain	
	Entropy	Purity	Entropy	Purity
LS (Baseline)	1.53	0.94	0.99	0.71
RSS + LS	1.39	0.95	0.93	0.72
IRSS + LS	1.40	0.95	0.96	0.72
RSS + IRSS + LS	**1.37**	**0.96**	**0.87**	**0.75**

**LS** represents the baseline system, in which the similarity computation is performed without any background knowledge. All of the other three systems use one or both types of knowledge considered in this study, namely, the relevant and irrelevant aspect sets.

It should be noted that we captured the background knowledge for each aspect from the COAE corpus mentioned in Section 4.1.1.

Compared with the baseline system **LS**, the system **RSS + LS**, in which the relevant aspect set is considered as a source of background knowledge, yields better results, with an *Entropy* of 1.39 and a *Purity* of 0.95 for camera domain and an *Entropy* of 0.93 and a *Purity* of 0.72 for mobile phone domain. This illustrates that the use of the relevant aspect set is effective in aspect clustering. Specifically, for an aspect *a*_*i*_, in addition to the knowledge of *a*_*i*_’s literal meaning, its relevant aspect set expanded from multiple sentence contexts is another meaningful dimension on which to measure the similarity between two aspects.

Moreover, the system **IRSS + LS**, in which the irrelevant aspect set is considered as a source of background knowledge, also yields superior results compared with **LS**, with an *Entropy* of 1.40 and a *Purity* of 0.95 for camera domain and an *Entropy* of 0.96 and a *Purity* of 0.72 for mobile phone domain. This proves that the irrelevant aspect set can also be treated as another set of important evidence for aspect clustering. Obviously, if aspect *a*_*i*_ appears in the irrelevant aspect set of aspect *a*_*j*_, then *a*_*i*_ and *a*_*j*_ cannot be grouped together. This background knowledge can naturally avoid incorrect grouping in a situation in which *a*_*i*_ and *a*_*j*_ are literally similar but do not in fact belong to the same group.

Based on the above results, the dimension of relevant aspect similarity (**RSS**) can be regarded as supplementary to the literal similarity (**LS**), and the dimension of irrelevant aspect similarity (**IRSS**) can be regarded as a filter for eliminating certain incorrect cases. Therefore, the two types of aspect relations **RSS** and **IRSS** are complementary to each other, and we combine them into the new system **RSS + IRSS + LS** based on the baseline **LS**. Table 2 shows that **RSS + IRSS + LS** performs best among all of the considered systems for the both domains, with an *Entropy* of 1.37 and a *Purity* of 0.96 for camera domain and an *Entropy* of 0.87 and a *Purity* of 0.75 for mobile phone domain.

All the above experimental results prove that the two types of background knowledge are effective for the product aspect clustering task, and our proposed method can be portable to different product domains.

#### Effectiveness of Capturing Background Knowledge from the Web

Although we can capture background knowledge from many domain-related sentences/reviews, the knowledge used to describe each aspect may be far from sufficient. Thus, in this paper, we resort to the Web to enrich the background knowledge available for each aspect. Table 3 presents a comparison of product aspect clustering results obtained when capturing background knowledge from the Web for both camera and mobile phone domains. Thus we can obtain three new systems, that is **Web + RSS + LS**, **Web + IRSS + LS** and **Web + RSS + IRSS + LS** respectively. For each comparative system, we used the method described in Section 2.3 to expand the available background knowledge.

**Table 3:** Comparison of the results of product aspect clustering when background knowledge is captured from the Web for both domains.

Method	Camera Domain		Mobile Phone Domain	
	Entropy	Purity	Entropy	Purity
LS (Baseline)	1.53	0.94	0.99	0.71
Web + RSS + LS	1.37	0.96	0.78	0.75
Web + IRSS + LS	1.38	0.96	0.92	0.73
Web + RSS + IRSS + LS	**1.28**	**0.98**	**0.62**	**0.81**

From Table 3, we can observe that for both domains with such expanded knowledge, each system achieves better performance compared with the results for the same system without knowledge from the Web presented in Table 2. This finding indicates that the cross-document background knowledge captured from the Web can provide richer evidence to describe each aspect. It further demonstrates that both types of knowledge, the relevant and irrelevant aspect sets, are effective when used in the aspect clustering task.

Moreover, we can obtain a similar conclusion from Table 3 as from Table 2, namely, that regardless of whether we capture the knowledge from the corpus or from the Web, the systems that include both the relevant and irrelevant sets yield better results than the system **LS** and the systems that combine both types of background knowledge perform the best.

Please note that in our method, how to learn the two types of background knowledge and how to learn more knowledge are the key parts. In other words, all the experiments are used to demonstrate the background knowledge is effective. Since the aspect clustering is a typical clustering task, we just try a simple clustering method—hierarchical clustering method to cluster the product aspects. We can certainly try other clustering methods in the future.

The complexity of the naive hierarchical clustering algorithm is *O*(*N*^3^) because we exhaustively scan the N*N matrix C for the largest similarity in each of *N* − 1 iterations, in which *N* represents the aspects that need to be processed [35]. In practice, we also evaluate the running time for our method. For camera domain, it takes us 2,654 seconds when clustering 1,189 aspects (that is, N = 1,189). And for mobile phone domain, it takes us 676 seconds when clustering 757 aspects (that is, N = 757). According to the time complexity *O*(*N*^3^), the running time of camera domain should be (1189)^3^/(757)^3^ ≈ 3.87 times of the running time of mobile phone domain. The actual running time of camera domain is 2,654/676 ≈ 3.93 times of the running time of mobile phone domain. We can obtain the same conclusion.

## Related Work

Aspect recognition is an important task in sentiment analysis, which has recently attracted considerable attention [1, 2]. Many efficient approaches have been developed for this task, most notably including rule-based methods [14, 20–22, 36] and supervised methods [23–25]. However, these works have focused only on aspect extraction rather than on aspect clustering. Because grouping synonymous aspects is critical for obtaining an effective opinion summary, several research works have used topic-model based methods [8, 11, 28, 37] to simultaneously extract and group product aspects. For example, Chen et al. [37] proposed the automatic learning of prior knowledge from a large number of reviews for the discovery of more coherent aspects. However, topic models always jointly model both aspects and sentiment words.

Our work is focused on the grouping of synonyms, in which words or phrases are grouped based on their similarities. There are two common types of similarity measures. One is based on pre-existing knowledge resources (such as thesauri or WordNet), and the other is based on distributional properties [38]. Several research works have focused on measures of the first type, and the method we propose in this paper can also be classed as being of the first type. For example, Liu et al. [18] grouped product features using WordNet synonyms, with poor results because of the limited background knowledge of WordNet. Zhai et al. [29] modeled the task as a semi-supervised learning problem using lexical similarity. However, their method required several manually selected seeds as input, which were random and therefore difficult to implement or experimentally reproduce. By contrast, our method is fully automatic, requiring no human intervention. Using the second type of approach, Zhai et al. [38] tested a method based on distributional similarity and found that this method did not perform well for the aspect clustering task. Therefore, in this paper, we focused solely on similarity measures of the first type, namely, those based on knowledge resources. Two types of background knowledge, namely, relevant aspect knowledge and irrelevant aspect knowledge, were captured from large numbers of reviews.

Our work is also related to Web mining based methods to some extent because we use Web resources to expand the background knowledge available for each aspect for use in this task. Web resources can be regarded as a large corpus. Thus, Web mining can provide additional and richer information or knowledge for many natural language processing tasks, such as machine translation [39], word sense disambiguation [40] and others. Inspired by this type of method, in this paper, we propose the automatic learning of background knowledge from the large amount of review data available on the Web to perform aspect clustering.

## Conclusion and Future Work

Aspect extraction and aspect clustering are both critical for practical applications of sentiment analysis and opinion mining. However, the existing research on the aspect clustering task is far from sufficient. In this paper, we propose a simple and effective unsupervised method that incorporates a large amount of cross-document background knowledge for use in this task.

The determination of how to properly cluster and generalize aspects with similar meanings but different representations is a significant obstacle. To address this problem, we attempt to learn background knowledge from many reviews as a supplement to the description of each aspect. We propose the use of two types of knowledge, relevant aspect sets and irrelevant aspect sets, for each aspect based on two types of relations between aspects. To capture additional knowledge, we can exploit information available on the Web.

Then, by incorporating the rich background knowledge gathered in this manner, we design several similarity computation methods and apply a commonly used hierarchical clustering method to group the extracted aspects. Experimental results obtained in both the camera and mobile phone domain demonstrate that a system that incorporates both types of background knowledge performs better than a system without them, indicating that the background knowledge learned in the proposed manner is useful for aspect clustering. Moreover, we observe that the knowledge obtained from the Web is richer than that obtained from a typical corpus. A system that uses knowledge from the Web significantly outperforms a system that uses only the knowledge available from a limited corpus.

With regard to future work, we intend to test several other clustering methods, such as topic model based methods, to evaluate the performance achievable using our learned background knowledge. We would also like to apply our aspect clustering approach to a practical sentiment analysis task to obtain a more accurate and complete summary of its performance.
